# Thermal decomposition and atmospheric pressure chemical ionization of alanine using ion mobility spectrometry and computational study

**DOI:** 10.1016/j.heliyon.2024.e39942

**Published:** 2024-10-29

**Authors:** Manijeh Tozihi, Hamed Bahrami, Masoumeh Garmabdashti

**Affiliations:** Department of Chemistry, University of Zanjan, Zanjan, 38791-45371, Iran

**Keywords:** Alanine, Ion mobility spectrometry, Decomposition, Protonation, Adduct ion, DFT

## Abstract

This study investigates the impact of thermal decomposition on the ion mobility spectrum of L-alanine using ion mobility spectrometry (IMS) and computational methods. By employing a post-injection delay system, we examined the evolution of ion peaks corresponding to thermal decomposition products and their interaction with protonated alanine. Experimental results revealed that the observed ion mobility spectra predominantly feature protonated isomers and adduct ions. Computational analysis using Density Functional Theory (DFT) predicted the thermodynamically favored structures and stabilities of these products. Findings indicate that protonation at the nitrogen site in alanine is more stable than at the oxygen site, and observed peaks correspond to protonated isomers and adducts formed with ammonium ions. Further investigations showed that thermal decomposition of alanine generates ammonia, contributing to the formation of new adduct ions. This research provides new insights into the behavior of amino acids under thermal conditions with implications for analytical chemistry and biochemistry.

## Highlights

• Thermal decomposition affects alanine's ion mobility spectrum.
• The release of ammonia from the thermal decomposition of alanine is confirmed.
• The alanine decomposition fragment contributes to the formation of new adduct ions.
• DFT calculation identifies stable structures of product ions.

## Graphical abstract

**Figure:**
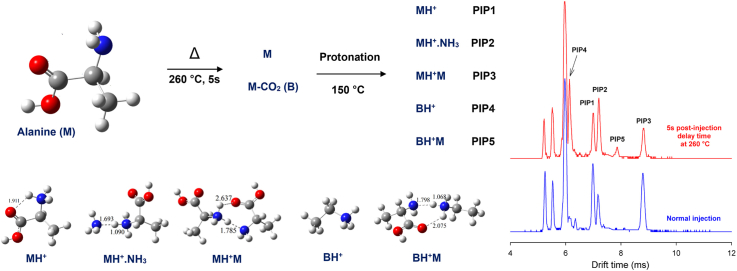

## 1 Introduction

Amino acids, as essential building blocks of proteins and vital biomolecules, have attracted considerable attention in fields such as chemistry, biochemistry, and molecular biology. The study of their chemical and physical behaviors, particularly under specific temperature and pressure conditions, is crucial for understanding their roles in biological systems and various chemical processes [1]. Thermal decomposition of amino acids can provide critical insights into their stability and decomposition pathways, which are essential for applications in biochemistry and materials science [2].

Ion mobility spectrometry (IMS) is a powerful technique for studying the chemical ionization behavior, fragmentation patterns and chemical properties of the molecules. The chemical ionization especially useful for analyzing small biomolecules, such as amino acids. IMS, in particular, has been widely used for the detection and differentiation of ions in gas-phase environments due to its high sensitivity and resolution under atmospheric pressure conditions. IMS separates ions based on their mobility in a drift tube under the influence of an electric field. IMS offers the capability to resolve ions based on their size, shape, and charge state, providing detailed insights into the ionization and fragmentation behavior of compounds [3]. IMS is a fast, sensitive, low-cost, and derivative-free technique used for the separation and detection of amino acids based on their mobilities [4,5].

Alanine is the simplest non-essential amino acid after glycine. It is the second most abundant amino acid in the blood and plays an essential role in metabolism after glutamine [6]. Understanding its physical and chemical behavior, particularly under specific conditions such as thermal decomposition and chemical ionization, is crucial for gaining deeper insights into its biochemical and chemical reactions.

Corona discharge ion mobility spectrometry (CD-IMS) in positive mode has been successfully used to separate and analyze L-alanine and sarcosine [7]. Sarcosine is proposed as a biomarker in the progression of prostate cancer. It has been specified that the ratio of sarcosine/alanine is high in urine derived from prostate cancer patients [8]. Differential ion mobility spectrometry (DIMS) coupling with tandem mass (MS/MS) spectrometry activated by resonant absorption of IR light (infrared multiple photon dissociation (IRMPD)) was used to separate sarcosine, α-alanine, and β-alanine, which are three isomers with the same mass [9]. IMS coupling with tandem mass spectrometry (IMS-MS/MS) has been applied to separate isomeric metabolites in human blood [10], and isobars and isomers of small molecules [11].

Thermal decomposition is a fundamental process in the study of amino acids, providing crucial insights into their stability, fragmentation mechanisms, and the formation of molecular ions under high-temperature conditions. Amino acids have been extensively studied due to their relevance in biological systems and their relatively simple structure, making them ideal candidates for thermal decomposition studies [[12], [13], [14], [15], [16], [17], [18]]. Alanine, is often used as a model compound to explore broader thermal degradation processes of peptides and protein [19]. Rates of thermal decomposition of alanine is described by the equation of first order reaction in the temperature range 200–300 °C [20]. Recently, Zengh et al. investigate the hydrothermal decomposition of alanine and studied the effects of various reaction conditions such as time, temperature, and material concentration on reaction routes [21].

Understanding the thermal behavior of alanine has implications for protein chemistry, enzymatic reactions, and the development of analytical techniques such as ion mobility spectrometry (IMS) and mass spectrometry [22]. The thermal decomposition of amino acids, such as alanine, is of significant interest due to its implications in biochemical processes and mass spectrometry-based analytical methods. In IMS, the decomposition of alanine can lead to the formation of distinct product ions, which provide insight into its structural and chemical properties.

Alanine, in addition to its aforementioned significance, is a suitable candidate for the investigation of ionization and thermal decomposition pathway due to its well-defined structural and chemical characteristics. L-alanine and all other natural amino acids, because of the intramolecular hydrogen bonds, exhibit flexible structures with low barriers for conformational isomerization [23,24]. Combined experimental and theoretical studies have been applied on the structures of free alanine that have given the structure for the lowest-energy conformer of the neutral alanine [23,25,26].

In recent years, computational chemistry has become an indispensable tool in various scientific fields, including biochemistry [27], and drug discovery [28], materials science [29], and optical technology [30]. Methods such as density functional theory (DFT) provide deep insights into molecular structures, stability, electronic and optical properties [31,32], reaction pathway [33], decomposition mechanisms [17], and thermodynamic properties with remarkable accuracy. In the study of thermal decomposition and ionization processes, computational approaches provide valuable insights into the formation of product ions and the stability of them.

This study explores the effect of thermal decomposition on the ion mobility spectrum of alanine, particularly focusing on the formation of product ions and the behavior of adduct species during the ionization process. By employing a post-injection delay time system, we investigate the evolution of ion peaks corresponding to thermal decomposition products and their interaction with protonated alanine. Additionally, DFT method is utilized to predict the thermodynamically favored products and provide a deeper understanding of alanine decomposition. The findings of this research contribute to a broader understanding of amino acid behavior in thermal environments, with potential applications in analytical chemistry and biochemistry.

## 2 Methods

### 2.1 Experimental

In this work, the commercial ion mobility spectrometer (model: IMS-300) made by TOF Tech. Pars. Co. (Isfahan, Iran) equipped with a corona discharge (CD) ion source in positive polarity, was used for all the experiments. The IMS cell consists of an ionization region a drift tube, separated by a Bradbury-Nielson shutter grid with an opening time of 30 μs in 20 ms intervals. A voltage of 8 kV was applied to the IMS cell to provide a uniform electric field of 500 V cm^−1^. The temperature of the injection port was set at 260 °C (maximum temperature of the region) to increase the evaporation efficiency of the sample. The drift tube temperature can be adjusted up to 200 °C controlled by a thermostat within ±2 °C. The experiments were carried out at 150 °C. Purified nitrogen gas was used as both the carrier and drift gases, with flow rates of 800 and 500 ml min^−1^, respectively. The drift tube operated at ambient pressure (1015 mbar). Alanine was purchased from Merck, was used in solid form and loaded into the injection port using an appropriate solid probe. The sample was vaporized in the injection port and then transferred to the ionization region via the carrier gas for gas phase ionization. Since the intensity of the reactant and product ion (PI) peaks changes over the injection period, the spectrum was presented at the highest intensity obtained at each point along the drift time axis through the entire sample introduction time. A post-injection delay time system was employed to study the thermal decomposition of alanine. In this system, a delay (1–60 s) was applied between the sample injection and its vapor introduction toward the ionization region.

### 2.2 Computational

Structures of neutral L-alanine, its decomposition products, and its ionic clusters-including reactant ion adducts of alanine and proton-bound dimers-were fully optimized using Density Functional Theory (DFT). DFT calculations were performed using the B3LYP hybrid functional [34]. The B3LYP functional combines Becke's three-parameter exchange functional [35] with the Lee-Yang-Parr correlation functional [34], incorporating a portion of Hartree-Fock exchange to improve the accuracy of electronic structure predictions [36]. This functional is widely used due to its balance between computational efficiency and accuracy, making it suitable for various applications in computational chemistry [37]. The B3LYP functional was used with the 6–311++G(d,p) Pople basis set [38], which includes both diffuse and polarization functions. For cluster ions, counterpoise calculations were performed to correct for basis set superposition errors (BSSE). To compute proton affinity (PA) and gas phase basicity (GPB), isomers of protonated alanine were optimized. Additionally, frequency calculations were carried out at the same level of theory. The PA and The GPB of a molecule, M, are defined as -ΔH° and – ΔG° of the following reaction, respectively.

(1) M(g) + H^+^(g) → MH^+^(g)

The protonation reaction is in the gas phase and 298 K. It should be mentioned that all calculations were carried out by Gaussian 09 package [39].

## 3 Results and discussions

### 3.1 Background ion mobility spectrum

**Fig. 1:**
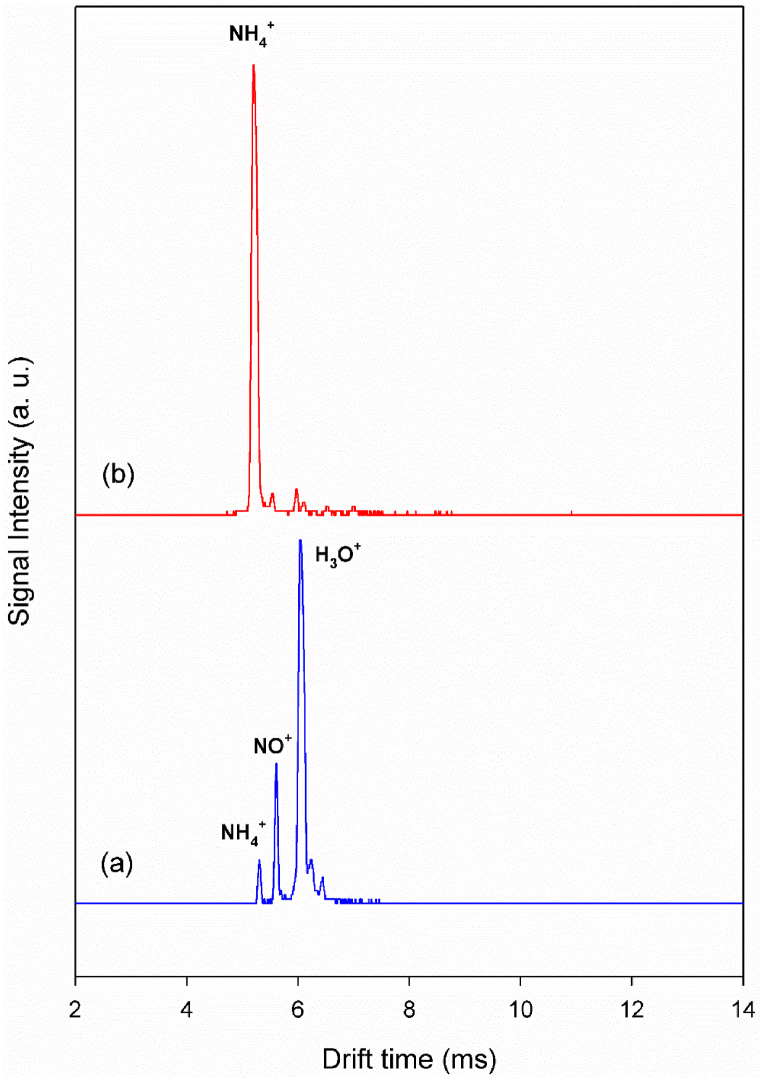
Background ion mobility spectrum at a cell temperature of 150 °C, (a) hydronium reactant ions, (b) ammonium reactant ions.

Fig. 1a shows the background ion mobility spectrum using a corona discharge ionization source in a nitrogen atmosphere at the cell temperature of 150 °C. The spectrum features three peaks at drift times of 5.3, 5.6, and 6.0 ms, corresponding to the hydrated NH_4_^+^, NO^+^, and H_3_O^+^ species, respectively, as the primary reactant ions (PRIs). As seen in this figure, the most intense peak is related to the hydrated hydronium ions, which are the dominant reactant ions. When ammonia enters the ionization region as a dopant, hydronium ions are converted into ammonium ions through a proton transfer reaction. Consequently, in the presence of a sufficient quantity of dopant, the dominant reactant ions will be ammonium ions (Fig. 1b).

### 3.2 Chemical ionization of alanine

**Fig. 2:**
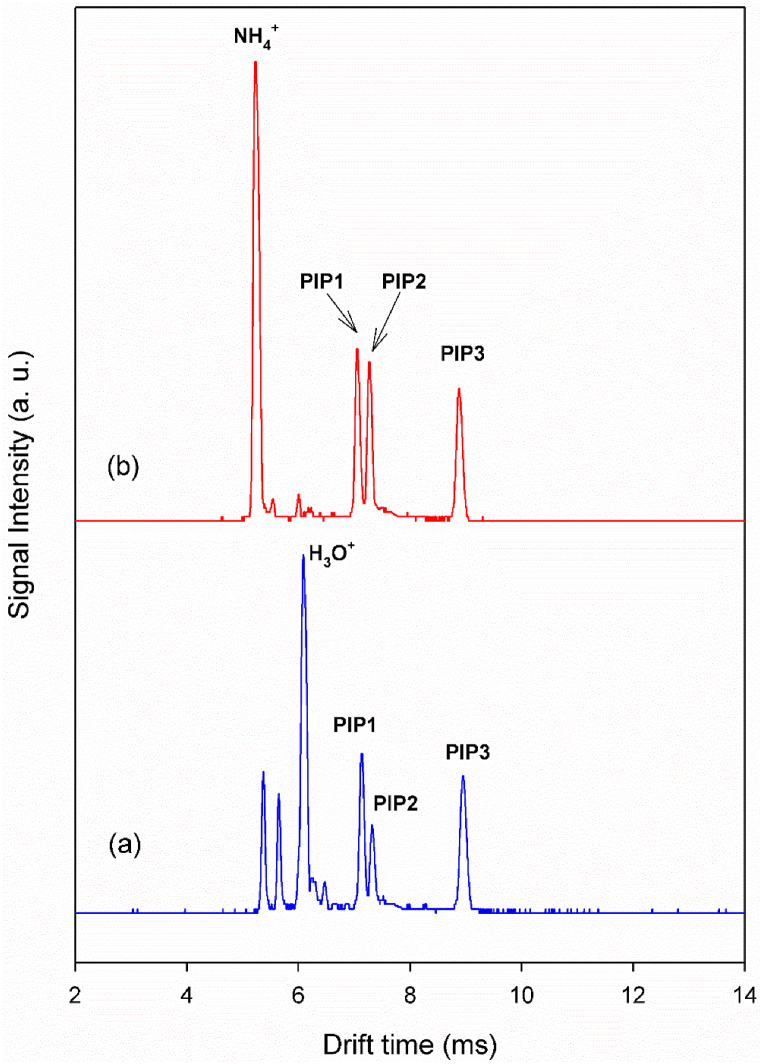
Ion mobility spectrum of alanine via CD ionization in nitrogen at the cell temperature of 150 °C, (a) with hydronium reactant ions, (b) with ammonium reactant ions.

In the process of chemical ionization of alanine, the sample is ionized in the ionization region through collisions between vaporized alanine molecules and reactant ions. Specifically, ionization occurs when the vaporized alanine interacts with hydronium ion in the ionization chamber, resulting in the formation of product ions [3]. The ion mobility spectrum of alanine with hydronium as the reactant ions was recorded at a cell temperature of 150 °C, as shown in Fig. 2a. At this temperature, in addition to the reactant ion peaks, three product ion peaks (PIPs) were observed for alanine. A similar pattern was seen in the ion mobility spectrum of alanine obtained with ammonium reactant ions, except that the relative intensity of PIP2 increased by approximately twofold. (Fig. 2b).

**Fig. 3:**
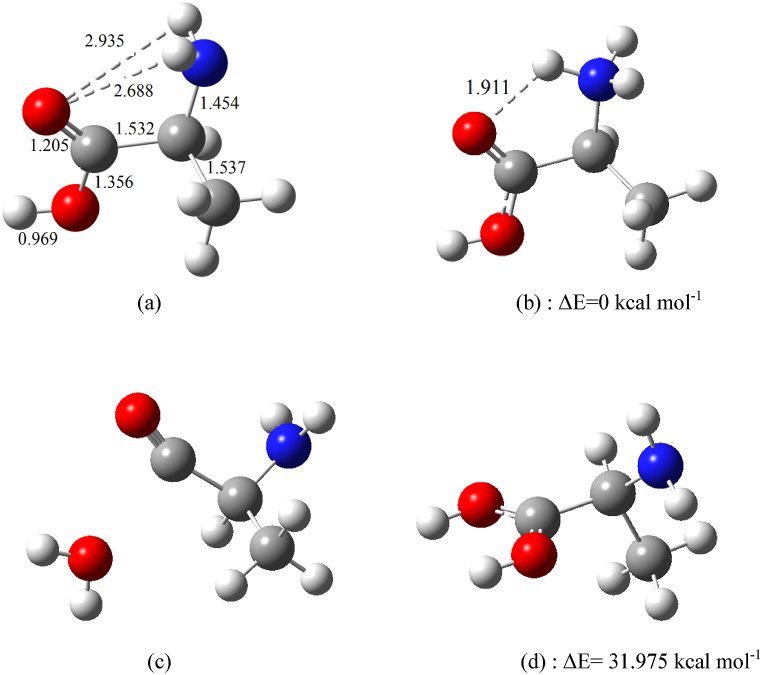
Optimized structures of alanine and its protomers at the B3LYP/6–311++G(d,p) level of theory; (a): neutral alanine, (b): protonated alanine at the N site, (c): protonation at the O site in OH group leading to the elimination of H_2_O, (d): protonated alanine at the O site in the CO group. The relative stability of protonated structures (**b** & **d**) is reported in kcal mol^−1^, and the bond length are in A°.

Alanine has two proton acceptor sites (N and O atoms), so PIPs 1 and 2 may correspond to protomers (protonated isomers) of alanine. To evaluate the accuracy of this prediction and interpret the observed peaks in the ion mobility spectrum, the protonation of alanine was investigated theoretically. Due to the presence of an acidic functional group (COOH) and an amino functional group (NH_2_), L-alanine as an amino acid, exhibits various intramolecular hydrogen bonds. Consequently, alanine has several conformers. As mentioned earlier, due to the intramolecular hydrogen bonds, alanine exhibits various conformers that have been widely studied from 1994 [40] to 2010 [24]. The following conformation shown in Fig. 3**a** is known as the most stable structure for the alanine molecule at the B3LYP/6–311++G(d,p) level of theory and was used in this work [41].

L-alanine, with the conformation shown in Fig. 3**a**, was protonated at the N and O sites. The optimized structures of these protonated forms are depicted in Fig. 3. After optimization of the initial protonated structure at the N site, the proton remains on the nitrogen atom (see Fig. 3**b**). Protonation of the OH group leads to the elimination of a water molecule (**c** in Fig. 3). Protonation of the CO group in trans position relative to the OH group causes the proton to migrate to the N atom during optimization, forming isomer **b** (Fig. 3). Conversely, protonation of the CO group in the cis position relative to the OH group results in a different isomer of protonated alanine (**d** in Fig. 3). The relative stability of the protonated structures (**b** & **d**) is shown in Fig. 3. The proton affinities (Pas) and gas-phase basicities (GPBs) of alanine for forming protonated isomer **b** and **d** were calculated at the B3LYP/6–311++G(d,p) level and are summarized in Table 1. The PA of alanine for protonation at the O site is higher than that of H_2_O (165 kcal mol^−1^) [42]. This indicates that protonation of the O site of alanine in the presence of H_3_O^+^ reactant ions is thermodynamically favorable. The calculated PA and GPB of alanine for protonation at the N site (Table 1) are higher than those for protonation at the O site, consistent with previous studies that reported the N is a stronger proton acceptor than the O site [43,44]. The calculated PA and GPB for the nitrogen (N) site in alanine closely match both experimental data [45] and other computational results [46] listed in Table 1. This consistency across sources validates our results and supports the reliability of our computational approach. The close match agreement with established values reinforces the robustness and precision of our findings.

**Table 1:** Proton affinity (PA) and gas-phase basicity (GPB) of alanine (in kcal mol^−1^) for protonation at the N and O (CO group) sites, calculated using the B3LYP functional and 6–311++G(d,p) basis set, in the gas phase and 298 K.

	Alanine (N atom)	Alanine (O of carbonyl group)	computational	Experimental
PA	215.270	185.182	216.80 [46]	215.6 [45]g
GPB	207.747	178.171	207.07 [46]	207.7 [45]

The relative energies of the protonated isomers (**b** and **d**) indicate that isomer **b**, where the N atom is the proton acceptor, is more stable than the isomer **d** where the O atom is the acceptor. The energy difference between the N-protonated and O-protonated isomers is significant, making the relative abundance of the O-protonated isomers **d** is too low to be observed in the ion mobility spectrum. On the other hand, PIP2 observed in the presence of ammonium (Fig. 2b), cannot be related to the O-protonated isomer because the PA of alanine at the O site is lower than that of NH_3_. Thus, the oxygen site of alanine has a limited chance of competing to receive the proton. Therefore, the observed PIPs 1 and 2 in the alanine ion mobility spectra are not due to the protonation at different sites (nitrogen and oxygen).

The PIP1 with the highest intensity in Fig. 2 (a & b) is attributed to the N-protonated isomer **b**. Notably, PIP2 cannot be assigned to a carbocation produced by water elimination from protonated alanine (protonated at the OH group in Fig. 2**c**) since PIP2 appears at a higher drift time, suggesting it is a heavier or bulkier ionic species than protonated alanine. Therefore, PIP2 is likely an adduct ion (M.NH_4_^+^ or MH^+^.NH_3_). Previous studies have confirmed ammonium attachment [47,48]. To identify the source of PIP2, theoretical investigation into ammonium attachment to alanine and ammonia attachment to protonated alanine were conducted.

The interaction between neutral alanine and ammonium ions may lead to the formation of MH^+^.NH_3_ and M.NH_4_^+^ clusters, according to the following reactions:

(2) M + NH_4_^+^ → MH^+^.NH_3_

(3) M + NH_4_^+^ → M.NH_4_^+^

**Fig. 4:**
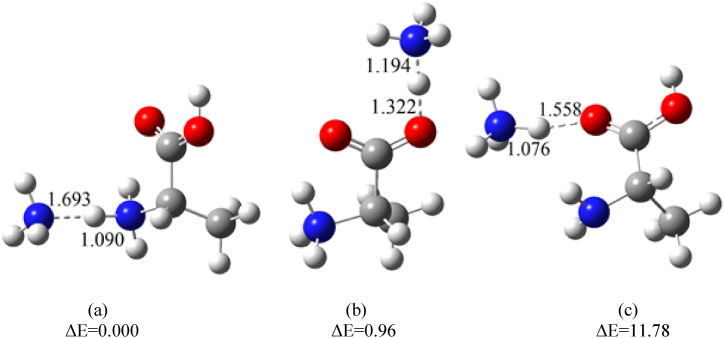
Optimized structures of adduct ions (MH^+^.NH_3_ & M.NH_4_^+^) at the B3LYP/6–311++G(d,p) level of theory; Relative energies (ΔE) are in kcal mol^−1^.

The interaction of alanine with ammonium ions was examined from different perspectives, with the optimized clusters shown in Fig. 4. When NH_4_^+^ approaches the nitrogen atom of alanine, higher proton affinity of alanine causes the proton from NH_4_^+^ transfers to the alanine molecule, forming isomers **a** in Fig. 4. Additionally, this isomer can be formed from the interaction between protonated alanine and an ammonia molecule.

(4) MH^+^ + NH_3_ → MH^+^.NH_3_

In structure **b**, the ammonia molecule forms a hydrogen bond with the OH group of protonated alanine. When NH_4_^+^ approaches the CO group in neutral alanine, the hydrogen bond between O of the carbonyl group and the hydrogen of NH_4_^+^ leads to the formation of the M.NH_4_^+^ cluster (isomer **c** in Fig. 4). Thus, when NH_4_^+^ interacts with neutral alanine, both MH^+^.NH_3_ and NH_4_^+^ attachment (M.NH_4_^+^) occur.

The relative electronic energies obtained at the B3LYP/6–311++G(d,p) level, shown in Fig. 4, compare the stabilities of different adduct ions. It is evident that there is no significant difference in the stability between isomers **a** and **b**, while isomer **c** is relatively unstable about 11 kcal mol^−1^. Consequently, the first two clusters in Fig. 4 are the primary contributors to the appearance PIP2 in the ion mobility spectrum of alanine (Fig. 2).

The ΔH° and ΔG° values for the formation of the related adducts were calculated using the B3LYP method and the e6-311++G(d,p) basis sets. The results are summarized in Table 2, with BSSE corrected values shown in parentheses. Negative ΔG° values indicate that the formation of adduct ions (MH^+^.NH_3_ and M.NH_4_^+^) is thermodynamically favorable.

**Table 2:** Calculated ΔH° and ΔG° values for the formation of adduct ions in the gas phase at 298 K. Values in parenthesis are corrected for basis set superposition errors (BSSE).

Reaction	ΔH° (kcal mol^−1^)	ΔG°(kcal mol^−1^)
M + NH_4_^+^ → MH^+^.NH_3_-a	−33.172 (−32.091)	−23.918 (−22.838)
M + NH_4_^+^ → MH^+^.NH_3_-c (M.NH_4_^+^)	−22.710 (−22.351)	−14.387 (−14.033)

MH ^+^+ NH_3_ → MH^+^.NH_3_-a	−21.412 (−20.331)	−13.229 (−12.149)
MH ^+^+ NH_3_ → MH^+^.NH_3_-b	−23.888 (−22.527)	−14.920 (−13.560)

M + MH^+^ → M.MH^+^_a	−25.220 (−24.139)	−14.494 (−13.413)
M + MH^+^ → M.MH^+^_b	−8.393 (−7.727)	−0.859 (−0.526)
M + MH^+^ → M.MH^+^_c	−17.858 (−17.325)	−8.817 (−8.284)

As seen in Fig. 2a, PIP2 appears with significant intensity when the dominant reactant ion is hydronium. In the absence of a dopant, there are two potential sources of ammonium ions. First, ammonium ions that are continuously produced in the source, and second, ammonia generated from the thermal decomposition of alanine. This excess ammonia is converted into NH_4_^+^ ions, which the interact with the neutral amino acid to form M.NH_4_^+^ or MH^+.^NH_3_. To verify this claim, a post-injection delay time system was used as discussed in the following section.

**Fig. 5:**
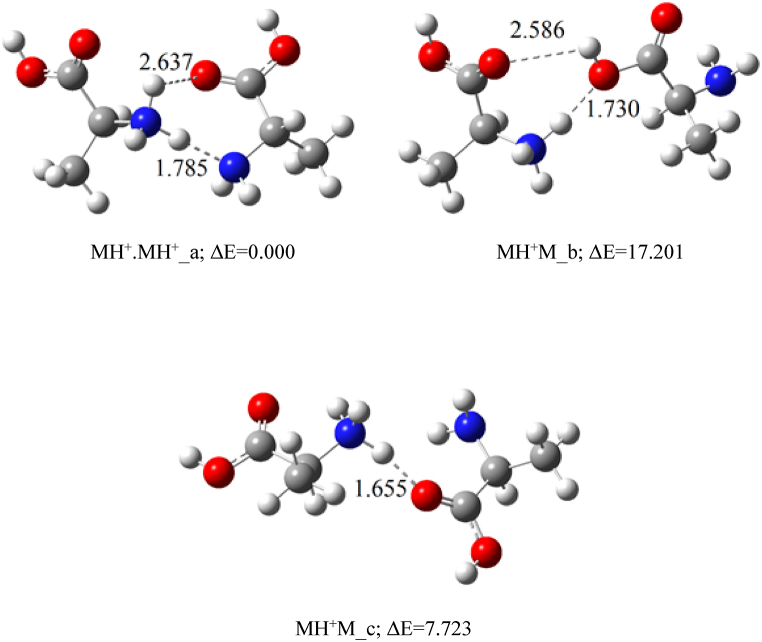
Optimized structures of the MH^+^.M cluster at the B3LYP/6–311++G(d,p) level of theory. Relative energies (ΔE) are shown in kcal mol^−1^.

In the IMS spectra of alanine, PIP3 appears with the longest drift time among PIPs. It is suggested that PIP3 corresponds to a proton-bond dimer of alanine. Fig. 5 shows the optimized structure for possible isomers of this dimer of alanine. Fig. 5 shows the optimized structures for possible isomers of this dimer at the specified level of theory. The relative energies presented in Fig. 5 indicate that isomer **a**, with two hydrogen bonds (NH…N and NH…O), is the most stable. Despite having only one hydrogen bond (NH…O), isomer **c** is more stable than isomer **b**, which has two hydrogen bonds (NH…O and O…HO).

The ΔH° and ΔG° values for the formation of all proton-bonded dimer isomers of alanine were calculated at the B3LYP/6–311++G(d,p) level of theory, with BSSE correction applied. The data in Table 2 indicate that the formation of isomers **a** and **c** related to the MH^+^.M cluster is thermodynamically feasible. Isomer **a** is thermodynamically more favorable than isomer **c**, suggesting that isomer **a** likely contributes more to the formation of PIP3 compared to isomers **b** and **c**.

To confirm the nature of the ionic species responsible for PIPs 1, 2, and 3, the time evolution of peaks was investigated and is described below. Typically, the intensity of the RIPs is decreases immediately after sample injection and then returns to its original height as the sample departs. In contrast, the intensity of the PIPs initially increases to a maximum and then decreases to zero from injection to sample departure. The differing time evolution of PIP intensities allows for the identification of product ions by examining the behavior of these peaks over time.

**Fig. 6:**
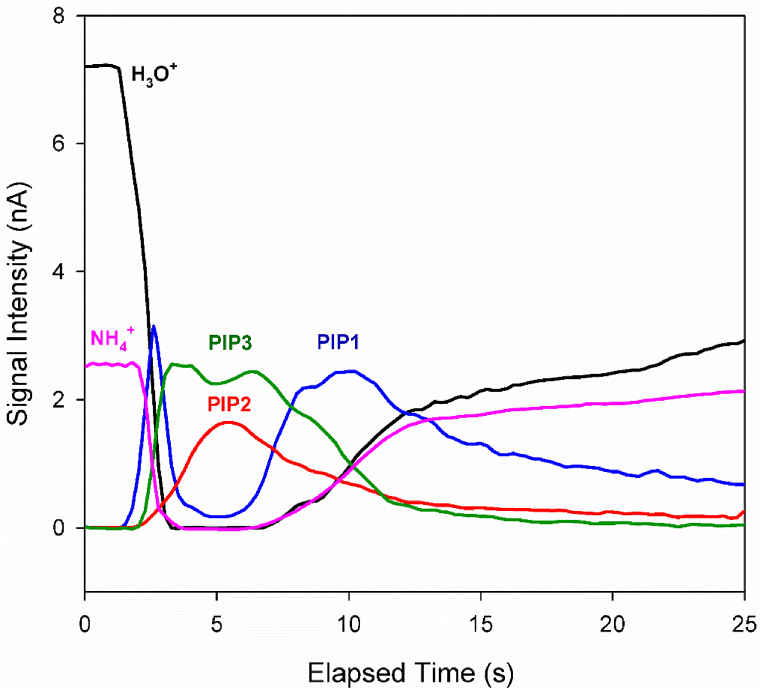
Time evolution of RIP and PIPs in the ion mobility spectrum of alanine via CD ionization in nitrogen at a cell temperature of 150 °C, with hydronium reactant ions.

Fig. 6 illustrates the variation in the intensities of alanine IMS peaks (from Fig. 2a) over time. When the sample enters the ionization region, the intensity of the RIP decreases rapidly to zero. In contrast, the intensity of PIP1 increases quickly to its maximum.

As the sample amount in the ionization region increases between 3 and 7 s, PIPs 2 and 3 reach their maximum values, while PIP1 temporarily decreases in intensity. The inverse relationship between the intensities of PIPs 2 and 3 and PIP1 confirms that alanine, whether in its neutral or protonated form, is consumed to produce the species responsible for PIPs 2 and 3. After 7 s, as the amount of sample molecules in the ionization region decreases, PIP1 becomes the dominant peak in the ion mobility spectra once again. Since the drift times of PIPs 2 and 3 are slightly higher than that of PIP1 (Fig. 2), these two peaks are likely adduct ions formed from the binding of ionic or neutral species to the alanine molecule or protonated alanine, respectively.

On the other hand, PIPs 2 and 3 appear when the sample amount is at its maximum in the ionization region. At this point, the number of sample molecules temporarily exceeds the number of reactant ions. Consequently, due to the competition for protons, the formation of adduct ions, including proton-bound dimers and reactant ion adducts, is likely.

In summary, PIP1, which is the most stable peak at low concentrations, can be attributed to protonated alanine. Given the drift time of PIP3 and the time evolution of the PIPs, PIP3 is assigned to the proton-bound dimer of alanine (MH⁺·M). PIP2, which has a slightly higher drift time than PIP1, is attributed to the ion adduct formed by the binding of ammonium ions present in the ionization region to the alanine molecule. According to Fig. 2, the relative intensity of PIP2 is higher in the presence of ammonium reactant ions. Additionally, it is established that ammonia is a product of the thermal decomposition of alanine [49,50]. Therefore, ammonium ions in the ionization region could originate from ammonia produced during the thermal decomposition of alanine. This provides a possible explanation for the presence of PIP2 in the spectrum obtained with hydronium reactant ions.

### 3.3 Thermal decomposition of alanine

**Fig. 7:**
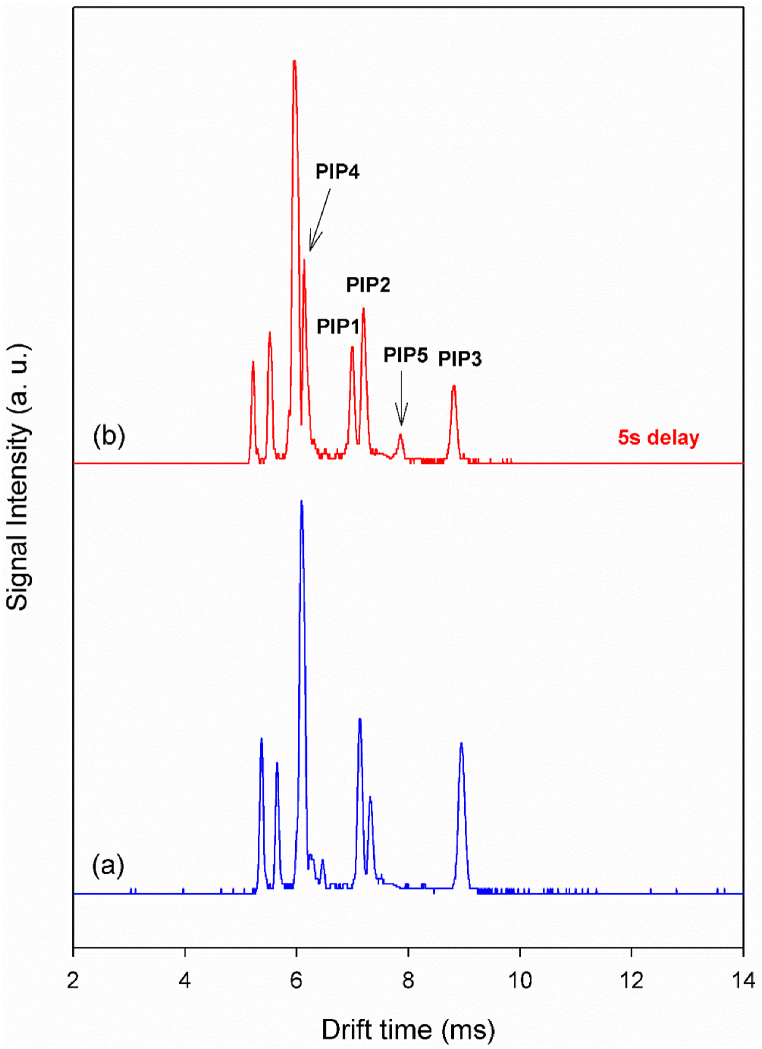
Ion mobility spectrum of alanine via CD ionization in nitrogen with hydronium reactant ions at a cell temperature of 150 °C: (a) normal injection and (b) 5-s post-injection delay time at 260 °C.

To investigate the effect of the thermal decomposition of alanine on the intensity of peaks in the ion mobility spectrum, a post-injection delay time system was employed. In this system, the vaporized sample is introduced into the ionization region after a specified delay time, allowing the sample to be exposed to heat for a controlled period in the injection chamber. The ion mobility spectrum of alanine obtained with a 5-s post-injection delay time at 260 °C is shown in Fig. 7 and compared with the spectrum resulting from a normal injection. As shown in Fig. 7, applying the post-injection delay time significantly increases the intensity of PIP2. This observation supports the claim that ammonia, produced from the thermal decomposition of alanine, contributes to the formation of an adduct ions.

On the other hand, applying the post-injection delay time leads to the appearance of two new product ions in the ion mobility spectrum, designated as PIP4 and PIP5. These product ions, formed by applying heat to alanine in the injection chamber, can be assigned to the fragment M − CO_2_ [49,50] and the adduct ion resulting from the coupling of this fragment with protonated alanine. The temporal evolution of the peaks, along with the predicted mass of the peaks, supports this claim.

**Fig. 8:**
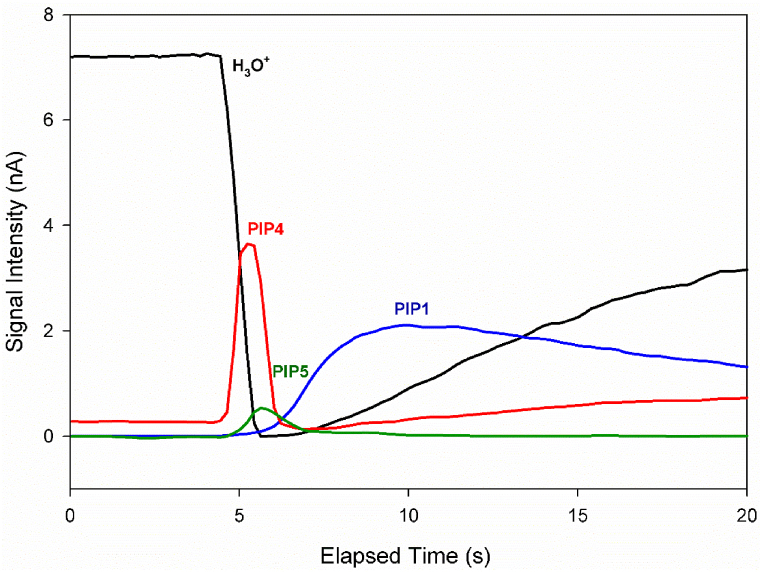
Time evolution of the hydronium RIP and PIPs in the ion mobility spectrum of alanine via CD ionization in nitrogen at a cell temperature of 150 °C, with a 5-s post-injection delay time at 260 °C.

Fig. 8 shows the change in the intensities of the hydronium RIP and several PIPs versus elapsed time in the ion mobility spectrum obtained with a 5-s delay time. It is evident that PIP4 appears immediately after the carrier gas flow starts, followed closely by PIP5. This observation indicates that the species forming PIP4 originate from the injection chamber. Additionally, as the intensity of PIP4 decreases, the intensity of PIP5 also diminishes, suggesting that the formation of PIP5 is dependent on the species responsible for PIP4.

**Fig. 9:**
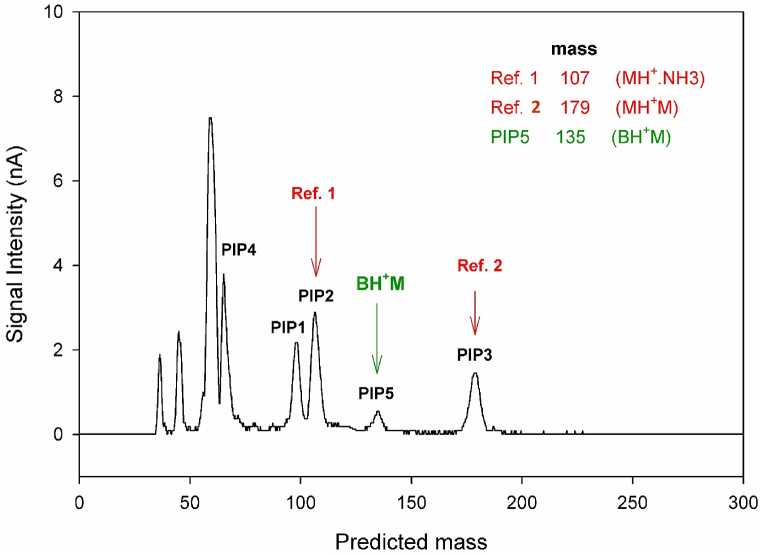
Mass converted spectrum of alanine obtained at injection port temperature of 260 °C with 5s post-injection delay time and cell temperature of 150 °C based on two standard masses.

The mass-drift time correlation equation, previously introduced by this research group and involving two standard masses, was used to predict the equivalent mass of PIP5 in the ion mobility spectrum shown in Fig. 7b. This method requires that the chemical nature of the reference ions be similar to that of the target ionic species. When the chemical similarity is maintained, the mass prediction is typically reliable within the range between the two reference masses. For this analysis, PIPs 2 and 3, which correspond to MH⁺·NH₄⁺ and MH⁺·M adduct ions with masses of 107 and 179, respectively, were selected as references. The ion mobility spectrum was then converted into a mass spectrum (Fig. 9). The predicted mass for PIP5 was found to be 135, which matches the mass of an adduct ion formed from the coupling of the M-CO₂ (B) fragment with protonated alanine.

**Fig. 10:**
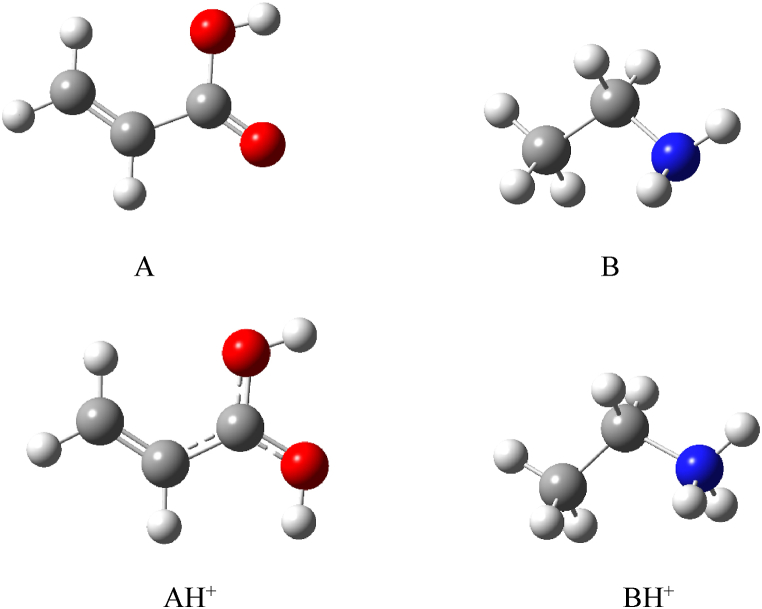
The optimized structures of the thermal decomposition products of alanine, M-NH₃ (A) and M-CO₂ (B), and their protonated forms (AH⁺ and BH⁺, respectively) at the B3LYP/6–311++G(d,p) level of theory.

Thermal decomposition products of alanine were evaluated using computational chemistry. It was found that the CH₂CHCOOH fragment (denoted as A) is produced from the thermal decomposition of alanine by releasing NH₃. With a proton affinity (PA) of 192 kcal mol⁻^1^, this compound is unlikely to succeed in protonation in competition with NH₃ and does not appear in the IMS spectrum. Another product, CH₃CH₂NH₂ (denoted as B in Fig. 10), results from the loss of CO₂. The compound B, with a proton affinity (PA) of 218 kcal mol⁻^1^, is more capable of proton absorption than NH₃ and H₂O, leading to the formation of BH⁺ in the presence of both ammonium and hydronium ions. The optimized structures of A, B, AH⁺, and BH⁺ are shown in Fig. 10. The ΔH° and ΔG° for the thermal decomposition of alanine and the protonation of its products were computed using the B3LYP method and are reported in Table 3. The ΔG° values indicate that the formation of B is thermodynamically more favorable than A. Therefore, PIP4 in Fig. 7 is attributed to BH⁺.

**Table 3:** The calculated ΔH° and ΔG° values for the reactions. The numbers in parentheses are corrected values for basis set superposition errors (BSSE).

Reaction	ΔH° (kcal mol^−1^)	ΔG° (kcal mol^−1^)
M → A + NH_3_	10.279	−0.405
M → B + CO_2_	−9.344	−19.571

A + H^+^ → AH^+^	−192.320	−184.679
B + H^+^ → BH^+^	−218.160	−210.604

M + BH^+^ → BH^+^M_a	−8.386 (−7.952)	−0.011 (0.423)
M + BH^+^ → BH^+^M _b	−17.708 (−17.27)	−8.952 (−8.523)
M + BH^+^ → BH^+^M _c	−20.432 (−19.569)	−11.124 (−10.261)
M + BH^+^ → BH^+^M _d	−26.653 (−25.781)	−16.106 (−15.234)
M + BH^+^ → BH^+^M _e	−29.295 (−26.126)	−19.129 (−15.694)

On the other hand, the other adduct ion is produced through the fallowing reactions:

(5) M + BH^+^ → BH^+^M

(6) MH^+^+B → BH^+^M

**Fig. 11:**
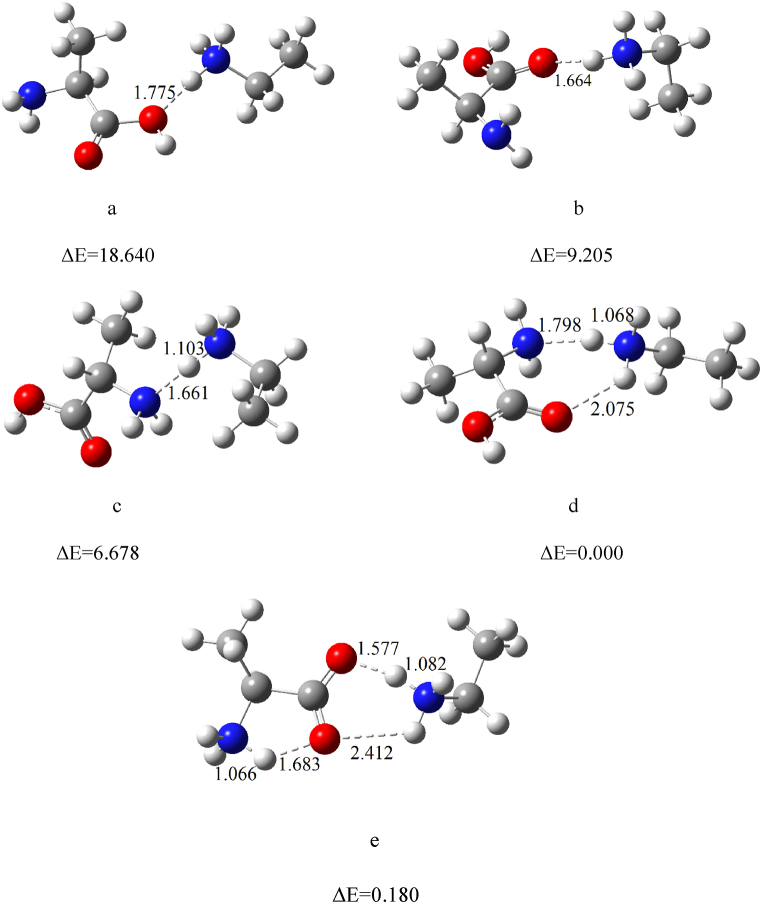
The optimized structures of BH ^+^ M cluster at the B3LYP/6–311++G(d,p) level of theory; relative energies (ΔE) are in kcal mol^−1^.

BH⁺M is known as an asymmetric proton-bound dimer, in contrast to MH⁺M, which is a symmetric proton-bound dimer. The optimized structures of BH⁺M, along with their relative energies, are shown in Fig. 11.

From Fig. 11, it is clear that isomers **d** and **e** are the most stable for the BH⁺M adduct ion and contribute significantly to the formation of PIP5. The negative values of ΔG° for the formation of these adduct ions confirm their presence in the IMS drift tube.

## 4 Conclusion

In this study, the effect of thermal decomposition on the ion mobility spectrum of alanine was investigated using ion mobility spectrometry and computational methods. The results revealed that chemical ionization of alanine (M) with hydronium and ammonium ions resulted in the formation of three major product ion peaks (PIPs) in the ion mobility spectrum, corresponding to the protonated L-alanine (MH^+^), adduct ion (MH^+^.NH_3_), and proton-bound dimer (MH^+^M), respectively. Computational analysis confirmed that protonation at the nitrogen site is thermodynamically more favorable than at the oxygen site, and the formation of product ions such as MH⁺·NH₃ and M·NH₄⁺ was validated. Furthermore, thermal treatment of alanine led to the production of ammonia and other decomposition products such as M − CO_2_ (B), resulting in the formation of new product ion peaks assigned to BH^+^ and BH^+^M. Subsequently, computational data indicated that the formation of these adduct ions was thermodynamically favorable. These findings provide deeper insights into alanine's behavior in thermal environments and contribute to improved interpretation of IMS data in complex biological systems.

## CRediT authorship contribution statement

**Manijeh Tozihi:** Writing – review & editing, Validation, Supervision, Software, Investigation, Formal analysis, Data curation, Conceptualization. **Hamed Bahrami:** Writing – review & editing, Validation, Investigation, Conceptualization. **Masoumeh Garmabdashti:** Investigation.

## Data availability statement

Data will be provided on request.

## Declaration of competing interest

The authors declare that they have no known competing financial interests or personal relationships that could have appeared to influence the work reported in this paper.
